# Changes in immune cell populations during acclimatization to high altitude

**DOI:** 10.14814/phy2.70024

**Published:** 2024-11-17

**Authors:** Kathy Pham, Abel Vargas, Shyleen Frost, Saheli Shah, Erica C. Heinrich

**Affiliations:** ^1^ Division of Biomedical Sciences, School of Medicine University of California Riverside Riverside California USA

**Keywords:** high altitude, inflammation, innate immune response, acute mountain sickness

## Abstract

The immune response to acute hypoxemia may play a critical role in high‐altitude acclimatization and adaptation. However, if not properly controlled, hypoxemia‐induced inflammation may exacerbate high‐altitude pathologies, such as acute mountain sickness (AMS), or other hypoxia‐related clinical conditions. Several studies report changes in immune cell subsets at high altitude. However, the mechanisms underlying these changes, and if these alterations are beneficial or maladaptive, remains unknown. To address this, we performed multiparameter flow cytometry on peripheral blood mononuclear cells (PBMCs) collected throughout 3 days of high‐altitude acclimatization in healthy sea‐level residents (*n* = 20). Additionally, we conducted in vitro stimulation assays to test if high‐altitude hypoxia exposure influences responses of immune cells to subsequent inflammatory stimuli. We found several immune populations were altered at high altitude, including monocytes, T cells, and B cells. Some changes in immune cell populations are potentially correlated with AMS incidence and severity. In vitro high‐altitude PBMC cultures stimulated with lipopolysaccharide (LPS) showed no changes in pro‐inflammatory cytokine production after 1 day at high‐altitude. However, by day three pro‐inflammatory cytokine production in response to LPS decreased significantly. These results indicate that high‐altitude exposure may initiate an inflammatory response that encompasses innate immune sensitization, with adaptive immune suppression following acclimatization.

## 1 INTRODUCTION

High‐altitude is a physiologically stressful environment due to reduced oxygen availability, low temperatures, low humidity, and other factors. Acute hypoxemia during travel to high altitude triggers a cascade of physiological responses to maintain oxygen homeostasis. Many of the physiological mechanisms that contribute to high‐altitude acclimatization and adaptation have been well described (Bartsch et al., 2002; Beall, 2006; Moore, 2017; Scheinfeldt et al., 2012; Simonson, 2015), such as the increase in minute ventilation and ventilatory chemosensitivity, cardiovascular changes such as increased red blood cell production and capillary density, as well as shifts in metabolic pathways. However, we are just beginning to understand the possible changes that occur within the immune system and associated inflammatory signaling networks during acute high‐altitude exposure.

Under typical conditions, immune cells are exposed to a wide range of oxygen tensions as they migrate from bone marrow to blood, and throughout the arterio‐venous circuit (Tsai et al., 2010). Furthermore, immune cells are mobilized to sites of inflammation or tissue injury, where hypoxia is an important feature that these cells must accommodate. As such, the molecular hypoxia‐ and inflammatory‐response pathways work in tandem and share significant crosstalk to mediate the response and resolution mechanisms for tissue injury or insult (Bandarra & Rocha, 2013; Corcoran & O'Neill, 2016; D'Ignazio et al., 2016; Görlach & Bonello, 2008; Pham et al., 2021). Immune cell adaptation to hypoxia is thought to be largely mediated by transcriptional activity of the hypoxia inducible factor (HIF) which is responsible for mobilizing hundreds of genes in response to oxygen limitation (Semenza, 1998, 2009). In addition to HIF being the master regulator of the cellular hypoxia response, HIF also plays a crucial role in immune cell metabolic function (Tao et al., 2015).

The crucial crosstalk between hypoxia and inflammation is a significant feature in critical illnesses, such as sepsis and acute respiratory distress syndrome. Indeed, hypoxemia may exacerbate inflammatory responses and subsequently worsen outcomes in these cases. High‐altitude exposure is a valuable method for examining the independent impact of hypoxemia on immune function and systemic inflammation in the absence of concomitant infection or injury and can provide unique insights into the role of hypoxia in immune system regulation in these pathologies. Previous research has investigated how acute high‐altitude hypoxia exposure influences the expression of inflammatory mediators in peripheral blood (Eltzschig, 2011; Faquin et al., 1992; Hartmann et al., 2000; Heinrich et al., 2018; Kiers et al., 2016; Lundeberg et al., 2018; Pham et al., 2022; Scholz et al., 2013), as well as immune cell adaptation and differentiation (Caldwell et al., 2001; Kiers et al., 2016; Tao et al., 2015; Zhu et al., 2022). While this work provides evidence that hypoxemia induced by high‐altitude exposure may contribute to acute increases in some pro‐inflammatory mediators under some ascent profiles, findings across studies remain inconclusive and it is unknown if this systemic inflammatory response is a consequence of, or a contributing factor to, the development of high‐altitude illnesses, such as acute mountain sickness (AMS), high‐altitude pulmonary edema (HAPE), high‐altitude pulmonary hypertension (HAPH), and high‐altitude cerebral edema (HACE) (Bärtsch & Swenson, 2013; Luks et al., 2017; Mallet et al., 2023). Furthermore, little is known regarding the mechanisms driving these changes in the inflammatory profile at high altitude, as well as potential changes in the immunological balance, and it is unclear if these changes are beneficial or maladaptive.

In the current study, we investigate the changes in the immune cell balance by characterizing peripheral blood mononuclear cells (PBMCs) during 3 days of acute high‐altitude hypoxia exposure in healthy sea‐level residents. Furthermore, since previous research has shown that acute high‐altitude exposure may upregulate the toll‐like receptor 4 (TLR4) signaling pathway (Pham et al., 2022), we aimed to determine if PBMCs collected at altitude are indeed more responsive to subsequent TLR4‐mediated inflammatory stimuli. We utilized flow cytometry to characterize PBMC populations and subpopulations at high altitude and conducted in vitro stimulation assays to determine if their function is altered during 3 days of high‐altitude exposure. We hypothesized that PBMCs would shift to a pro‐inflammatory phenotype upon initial acute high‐altitude hypoxia exposure. As a result, these cells would be sensitized to inflammatory stimuli and produce elevated pro‐inflammatory cytokine release when stimulated with a TLR4 ligand. As individuals acclimatize, we expected a shift to an anti‐inflammatory profile as complimentary physiological changes occur to improve oxygen delivery to tissue.

## 2 METHODS

### 2.1 Ethical approval

This study was approved by the University of California, Riverside Clinical Institutional Review Board (HS 22‐088). All participants were informed of the study's purpose and risks. Participants provided written informed consent in their native language (English). The work was conducted in accordance with the *Declaration of Helsinki*, except for registration in a database.

### 2.2 Participants

The study included 20 healthy participants (*N* = 7 women, 13 men) between 19 and 35 years of age. Participants were recruited by word of mouth and flyers on the UC Riverside campus. All participants reported no known history of cardiopulmonary disease or sleep disturbances, including obstructive sleep apnea, and displayed no abnormal findings on electrocardiogram (ECG) or pulmonary function testing. Mean age was 25 ± 7 years for women and 26 ± 6 years for men and mean BMI was 30 ± 5.4 kg/m^2^ for women and 31 ± 5.3 kg/m^2^ for men. Exclusion criteria included travel above 8000 feet within 1 month of the first measurements, a previous history of high‐altitude pulmonary or cerebral edema, current smoking, and pregnancy.

### 2.3 Experimental design and physiological measures

In the 2 weeks prior to ascent to high altitude, participants completed initial screening for eligibility at UC Riverside, located at approximately 400 m elevation (Riverside, CA, USA). Demographic information including age, height, weight, and blood pressure were collected. Participants also answered questions about their ancestral background (to determine presence of high‐altitude ancestry) and medical history including current medications. Participants then completed pulmonary function testing and ECG to verify absence of lung or heart disease.

Participants returned to UC Riverside in the early morning on the day of ascent. Baseline (sea‐level, SL) physiological measures were collected at this time, including blood pressure, resting pulse oximetry (SpO_2_), resting heart rate, and AMS scores via the 2018 Lake Louise scoring criteria with an experimenter asking participants each question (Roach et al., 2018). Depending on the score, participants were noted with mild AMS (AMS score 3–5 with headache) or moderate – severe AMS (AMS score 6+ with headache). Fasting blood samples were then collected via standard venipuncture procedures. Breakfast was provided to participants following blood sampling, prior to travel.

The group then traveled by car to Barcroft Station (3800 m elevation) in the White Mountain Research Center (Bishop, CA, USA) over a period of approximately 6.5 h. At the field station, fasting blood samples and morning measurements were collected each day within 1 h of waking and before 9 a.m. to keep timing consistent with sea level measures. Physiological measures and fasting blood samples were collected every morning for three consecutive days (HA1, HA2, and HA3). A 3‐day study was chosen since a majority of the acclimatization process occurs by the third day of exposure at this elevation, and a majority of AMS symptoms are alleviated by this time. Pulse oximetry and heart rate values were collected using a Nellcor N‐600 pulse oximeter (Medtronic, Minneapolis, MN, USA). Participants sat upright in a chair without their legs crossed and rested, breathing normally, for 3 min until values stabilized. Blood pressure measurements were collected in duplicate while participants rested in an upright seated position using a manual sphygmomanometer.

Participants abstained from taking anti‐inflammatory medications or other agents that may influence acclimatization, such as acetazolamide (Basaran et al., 2016). Participants were permitted to consume caffeine in moderation (one cup of coffee or tea) after completing morning measurements but were asked to abstain from caffeine after noon. Three meals per day were provided and participants did not complete any strenuous physical activity. Participants did not consume alcohol, and fluid intake was supervised to ensure participants remained hydrated.

### 2.4 Immune cell characterization

#### 2.4.1 Isolation and transport of PBMCs

Peripheral venous blood was collected in a 10 mL vacutainer tube containing EDTA (BD, Franklin Lakes, NJ, USA) and processed within 4 h of collection. Blood was diluted 1:1 with phosphate buffered saline (PBS). In a separate tube, an equal volume of Lymphoprep Density Gradient Medium (1.077 g/mL density) (StemCell, Seattle, WA, USA) was added. Blood was then slowly layered on top of the Lymphoprep. Tubes were centrifuged at 400×*g* for 30 min at room temperature, with slow acceleration and no brakes. PBMCs were carefully collected into a separate 15 mL tube with 8 mL of EasySep media (StemCell, Seattle, WA, USA). PBMCs were centrifuge at 400×*g* for 5 min and resuspended in 5 mL of EasySep media. The wash step was repeated twice. PBMCs were resuspended in 1.5 mL of freezing media (90% FBS, 10% DMSO), and aliquoted in 500 μL volumes. Aliquots were placed in a Mr. Frosty freezing container (ThermoFisher, Carlsbad, USA) and into a −80°C freezer overnight, then transferred to liquid nitrogen the following morning.

At high altitude, the same procedure was followed, with the following exceptions. Due to a lack of a −80°C freezer at high altitude, the Mr. Frosty containers were placed in a large Styrofoam box filled with dry ice overnight. Aliquots were transferred to a transportable liquid nitrogen dewar for transport to sea level. Due to logistical constraints and timing, PBMCs collected on Day 3 at altitude were first collected as buffy coat while at Barcroft Station, and then processed for PBMCs following the same protocol the following day at sea level. Separate experiments conducted in our lab to compare storage protocols revealed no significant differences in cell viability or inflammatory activity across these protocols.

When thawing PBMCs for downstream analyses, PBMCs were removed from liquid nitrogen and placed in a 37°C water bath for 30–45 s, or until a small ice crystal was left. Warmed media of 1 mL (RPMI 1640, 10% FBS, 100 U/mL Strep/Penicillin) was added in a dropwise manner to the tube, which was then transferred to a 15 mL falcon tube containing 5 mL of warmed media. The tubes were gently mixed by inverting, and then centrifuged at 330×*g* for 10 min at room temperature. PBMCs were then resuspended in 1 mL of warmed media and counted via hemocytometer for experiments.

#### 2.4.2 Flow cytometry for immune cell characterization

Paired PBMC samples from sea level, day 1 at high altitude, and day 3 at high altitude were stained and analyzed via flow cytometry, with sea level samples used as control. Cells were thawed, checked for viability, and counted with Trypan blue. 3.0 × 10^5^ PBMCs/100μL media from each sample were aliquoted for the experiment. PBMCs were then centrifuged at 330×*g* for 10 min and resuspended in 100 μL of a 1:20 dilution of Human TruStain FcX (Fc Receptor Blocking Solution) (BioLegend, San Diego, USA) in FACS buffer. Cells were stained as per manufacturer recommendations with a 1:400 dilution (5 μL of antibody per 1 million cells in 100 μL) using fluorescent antibodies: anti‐CD3 PerCP/Cyanine 5.5 (BioLegend, San Diego, USA; Clone OKT3), anti‐CD11b APC/Cyanine 7 (BioLegend, San Diego, USA; Clone M1/70), anti‐CD25 BV 420 (BioLegend, San Diego, USA; Clone BC96), anti‐CD45 BV 711 (BioLegend, San Diego, USA; Clone HI30), anti‐CD66b APC (BioLegend, San Diego, USA; Clone G10F5), anti‐CD56 (BioLegend, San Diego, USA; Clone HCD56), anti‐CD14 AlexaFluor 488 (BioLegend, San Diego, USA; Clone HCD14), anti‐CD163 BV 510 (BioLegend, San Diego, USA; Clone GHI/61), anti‐CD8 BV 785 (BioLegend, San Diego, USA; Clone SK1), anti‐CD16 PE (BioLegend, San Diego, USA; Clone 3G8), anti‐CD19 PerCP‐eFluor 710 (eBioscience, San Diego, USA; Clone HIB19), anti‐CD4 PE/Cyanine 5 (BioLegend, San Diego, USA; Clone A16A1), and anti‐HLA‐DR, DP, DQ (BD Biosciences, San Diego, USA; Clone Tu39). Antibody information is found in Table S1. After staining, PBMCs were fixed with 4% PFA for 10 min, washed and resuspended in 2 mL FACS buffer. Samples were analyzed using the NovoCyte Quanteon flow cytometer, NovoSampler Q, and NovoExpress Software. The gating strategy was modified from a previously published protocol using the same immune cell characterization panel for whole blood (Bergersen et al., 2023). An average of 1.0 × 10^5^ events were collected in total for analysis. Analysis was conducted using FlowJo software version 10.0. Proper compensation was determined using the Novocyte automatic compensation algorithm. We visually inspected fluorescence signals and manually optimized the automatic compensation by narrowing filters when required to avoid spillover. Fluorescence minus one (FMO) analyses were performed on PBMCs for this large immune panel to verify signal accuracy and adjust gating strategy accordingly.

#### 2.4.3 Flow cytometry for TLR4 surface expression analysis

In a separate panel, 1.0 × 10^5^ cells PBMCs from sea level (SL), first day (HA1) and third day (HA3) at altitude were stained with LIVE/DEAD Fixable Far Red Dead Cell Stain (Thermofisher, Carlsbad, USA) for 30 min at 4°C. After staining, cells were washed with FACS buffer, centrifuged, and resuspended in 100 μL of FACS buffer. Cells were stained with a 1:400 dilution using fluorescent antibodies: anti‐CD14 AlexaFluor488 (BioLegend, San Diego, USA; Clone HCD14) and anti‐TLR4 PE (BioLegend, San Diego, USA; Clone HTA125). Antibody information is found in Table S2. Samples were analyzed using the NovoCyte Quanteon flow cytometer, NovoSampler *Q*, and NovoExpress Software. An average of 1.0 × 10^5^ events were collected in total for analysis. Analysis was conducted using FlowJo software version 10.0.

#### 2.4.4 Inflammatory stimulation assays

PBMCs collected at sea level, as well as the first day and third day at altitude, were used for stimulation experiments. 1.0 × 10^5^ PBMCs at each timepoint (SL, HA1, and HA3) were used in the experiment. PBMCs were stimulated with 100 ng/mL LPS (*E*. *coli* Serotype (026: B6)) (Cat. #: L8274, Sigma‐Aldrich, Burlington, Massachusetts, USA) in a 37°C incubator under normoxic conditions (21% O_2_; 5% CO_2_) for 6 h. Following culture, samples were centrifuged at 330×*g* for 10 min, and supernatant was collected and stored at −80°C for ELISA analysis.

#### 2.4.5 ELISA analysis

According to manufacturer's instructions, the concentration of TNF‐α and IL‐6 in cell culture supernatants were analyzed using a TNF‐α (Catalog # 88‐7346‐88) and IL‐6 (Catalog # 88‐7066‐88) Human ELISA kit (Invitrogen, Carlsbad, USA). An automated microplate reader (Synergy Lx Multimode Reader) (Biotek, Seattle, Washington, USA) was used for the measurement of the optical density at 450 nm. The concentrations of each sample were detected based on optical density (OD) and the concentration of the standard.

### 2.5 Statistical analysis

Statistical analyses were conducted in R (version 4.1.0) (R Foundation) and GraphPad Prism v10.0. To identify changes in physiological variables and immune cell phenotypes at high altitude compared to baseline sea‐level measures, we used repeated measures ANOVA and post‐hoc pairwise *t*‐tests with Bonferroni corrections. To determine if changes in immune cell phenotype were associated with physiological measures at high altitude (SpO_2_, AMS Score), Pearson correlation coefficients and *p* values were obtained with the *rcorr* function from the *Hmisc* package in R. Data is presented throughout the paper as mean ± standard deviation or 95% confidence interval. Three participants did not have PBMCs collected for one or more days at high altitude due to blood collection complications and were excluded from immune cell characterization analysis. In the event of outliers in cell culture assays, they were removed via the ROUT method.

## 3 RESULTS

### 3.1 Physiological measures

Table 1 provides an overview of physiological measures at sea level and over 3 days of acclimatization to high altitude. On the first morning at high altitude (HA1), 8 of 20 subjects indicated mild AMS (AMS score 3–5 with headache), and 6 of 20 subjects indicated moderate – severe AMS (AMS score 6+ score with headache). By the third day at altitude, 5 of 20 subjects indicated mild AMS, and 3 of 20 subjects indicated moderate – severe AMS. SpO_2_ dropped by about 11 percent on the first day at high altitude and remained lower than sea‐level values throughout all 3 days at altitude. This was coupled with a 17‐point increase in heart rate on the first day at high altitude, which increased a further 9 points by day 3. There was no significant change in systolic or diastolic blood pressure at high altitude.

**TABLE 1:** Physiological measures at baseline and over 3 days at high altitude.

Variable	SL	HA 1	HA 2	HA 3	ANOVA *p*
P_sys_	121 ± 7.1	126 ± 9.5	128 ± 13	126 ± 11	0.177
P_dia_	79 ± 5.0	85 ± 7.1	85 ± 8.4	85 ± 8.7	0.059
HR	74 ± 8.4	91 ± 13*	90 ± 15*	100 ± 16***	<0.001
SpO_2_	95 ± 1.7	84 ± 4.3***	84 ± 3.2***	84 ± 4.1***	<0.001
AMS	0.5 ± 0.6	4.3 ± 2.7***	4.0 ± 2.4**	2.6 ± 2.5*	<0.001

*Note*: Variable units: P_sys_ and P_dia_ (mmHg); HR (bpm); SpO_2_ (%). Overall *p* values for repeated measures ANOVA are provided. Asterisks indicate significant differences from SL at *p* < 0.05 (*), *p* < 0.01 (**), and *p* < 0.001 (***) levels via post‐hoc pairwise comparisons with Bonferroni adjusted *p* values.

### 3.2 Acute high‐altitude exposure promotes a shift in immune cell populations throughout acclimatization

**FIGURE 1:**
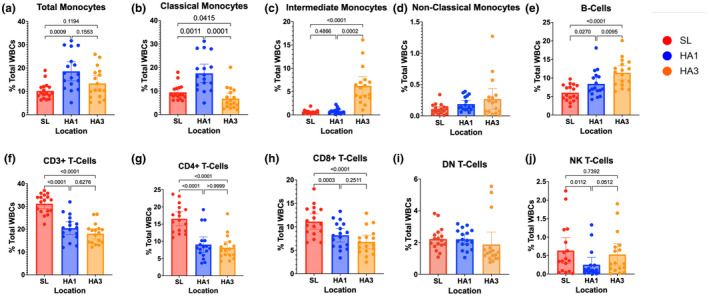
Immune cell population changes during 3 days of acute high‐altitude exposure. PBMCs collected at sea level (SL), as well as one (HA1) and three (HA3) days at high altitude. Plots represent changes in cell populations as a function of total white blood cells (WBCs). Populations include total monocyte (a) and monocyte subsets (classical (b), intermediate (c), non‐classical (d)), as well as B cells (e) and T‐Cell populations (Total CD3^+^ T cells (f), CD4^+^ T‐Cells (g), CD8^+^ T‐Cells (h), double negative (DN) T‐cells (i), and natural killer (NK) T‐cells (j)). Bar plots represent means and error bars represent 95% confidence intervals. Post‐hoc pairwise *t*‐test *p* values are provided for datasets showing significant main effects of location via one‐way repeated measures ANOVA.

**FIGURE 2:**
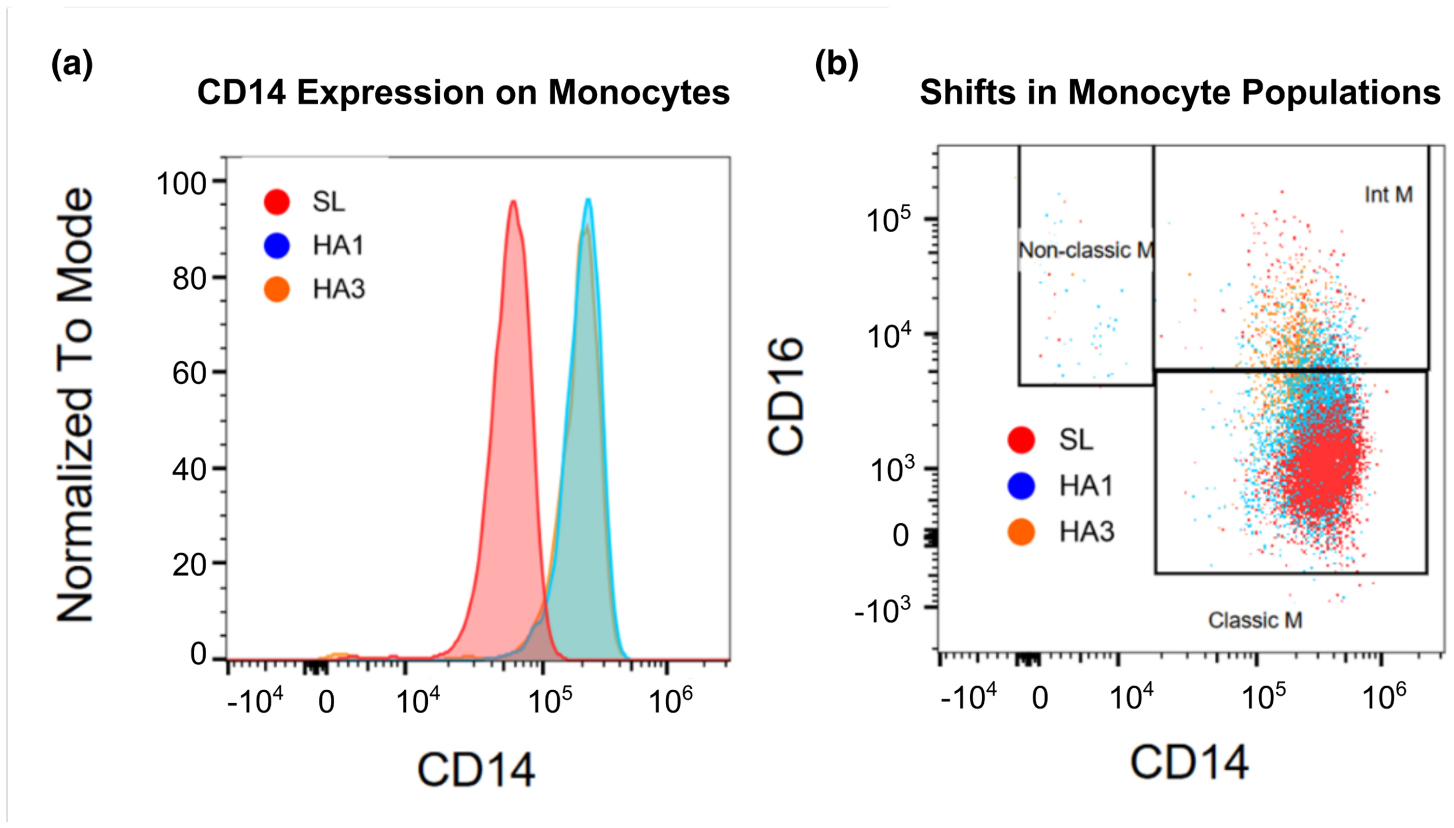
Representative sample data of CD14 surface expression on monocytes during 3 days at altitude. (a) CD14 surface expression intensity on monocytes over 3 days at altitude. (b) Monocyte subpopulation shifts over 3 days at high altitude show progressive increases in intermediate and non‐classical monocytes over time. Both plots provide representative data from one participant.

Immune cell subsets were quantified to identify changes in cell phenotypes over the course of 3 days at high altitude compared to sea level values (*n* = 17). Flow cytometry gating of PBMCs was performed to quantify innate and adaptive immune cells. The complete gating strategy is provided in Figure S1. Total monocyte frequency as a percentage of total white blood cells (% WBCs) was significantly elevated on the first day of acute high‐altitude exposure (following the night of arrival) compared to sea‐level values (Figure 1a), (main effect of location: *F*(1.4, 23.0) = 9.35, *p* = 0.003). Specifically, classical monocytes were significantly elevated on the first day at high altitude (Figure 1b, main effect of location: *F*(1.4, 22.2) = 23.71, *p* < 0.001). By the third day at high altitude, classical monocyte populations returned to baseline, while intermediate monocyte population continued to increase (Figure 1c, main effect of location: *F*(1.0, 16.5) = 30.41, *p* < 0.001). Although non‐classical monocytes show a trend for higher levels on the first day at high altitude, there was no main effect of location (Figure 1d,f (1.2, 18.8) = 3.05, *p* = 0.092). To complement this, shifts in monocyte subpopulations towards intermediate monocytes were observed following high altitude exposure, with a respective increase in CD14 surface expression (Figure 2). When analyzing based on frequency of total monocytes, classical monocyte subpopulation did not change on first day of altitude (*p* = 0.812) but was significantly reduced by the third day (*p* < 0.0001). Intermediate monocytes subpopulations also did not change after one day at altitude (*p* = 0.20) but was significantly higher by the third day (*p* < 0.0001) (Figure S3).

When analyzing B cell populations as a percentage of total WBCs, B cells were significantly elevated following one to 3 days (*p* = 0.0012) at high altitude (Figure 1e,f (2,32) = 21.25, *p* < 0.001).

T cell populations were analyzed as a percentage of total WBCs (Figure 1b). CD3^+^ T cells were significantly reduced on the first and third day of altitude (Figure 1f, main effect: *F*(1.4, 22.0) = 43.44, *p* < 0.001). Specifically, CD4^+^ and CD8^+^ T cells was significantly reduced on first and third day at high altitude (Figure 1g,h, main effects: *F*(1.5, 23.7) = 43.9, *p* < 0.001; *F*(1.5, 24.0) = 25.78, *p* < 0.001, respectively). There was no impact of altitude on DN T cells (CD3^+^ CD4^−^ CD8^−^ CD16^−^) (Figure 1f,i (1.3,21.3) = 1.06, *p* = 0.338). NK T cells were significantly reduced on the first day at altitude but recovered to baseline values by day 3 (Figure 1f,j (2,32) = 7.57, *p* = 0.002). These results demonstrate acute high‐altitude alters both innate and adaptive immune populations.

**FIGURE 3:**
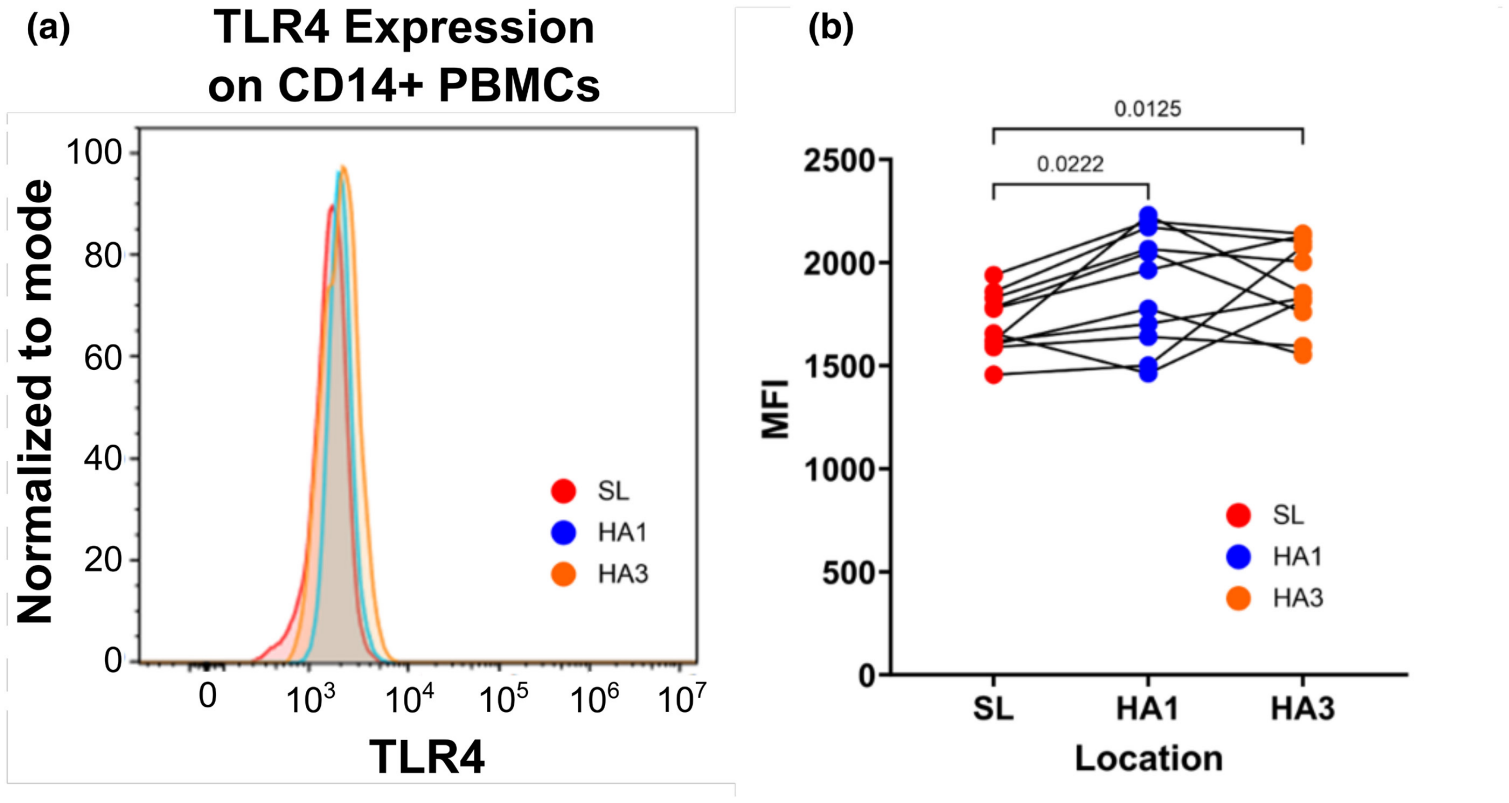
TLR4 surface expression on PBMCs during 3 days at high altitude. (a) Representative sample data of TLR4 surface expression intensity on CD14^+^ live PBMCs. Data shows a representative change in one participant. (b) MFI quantification of CD14^+^ TLR4^+^ surface expression on PBMCs measured via flow cytometry. Post‐hoc pairwise *t*‐test *p* values are provided after significant main effect of location via one‐way repeated measures ANOVA.

TLR4 expression on the surface of live CD14^+^ PBMCs collected at sea level and high altitude were quantified to measure effects of high altitude on the TLR4 signaling pathway, of which key components were previously found to have significant gene upregulation (Pham et al., 2022). Mean fluorescence intensity (MFI) of TLR4 was detected via flow cytometry (Figure S2). MFI of CD14^+^TLR4^+^ live PBMCs was significantly increased on day 1 at high altitude (*p* = 0.022) and was sustained on day 3 (*p* = 0.013) (Figure 3).

### 3.3 Inflammatory sensitivity of PBMCs collected at high altitude

**FIGURE 4:**
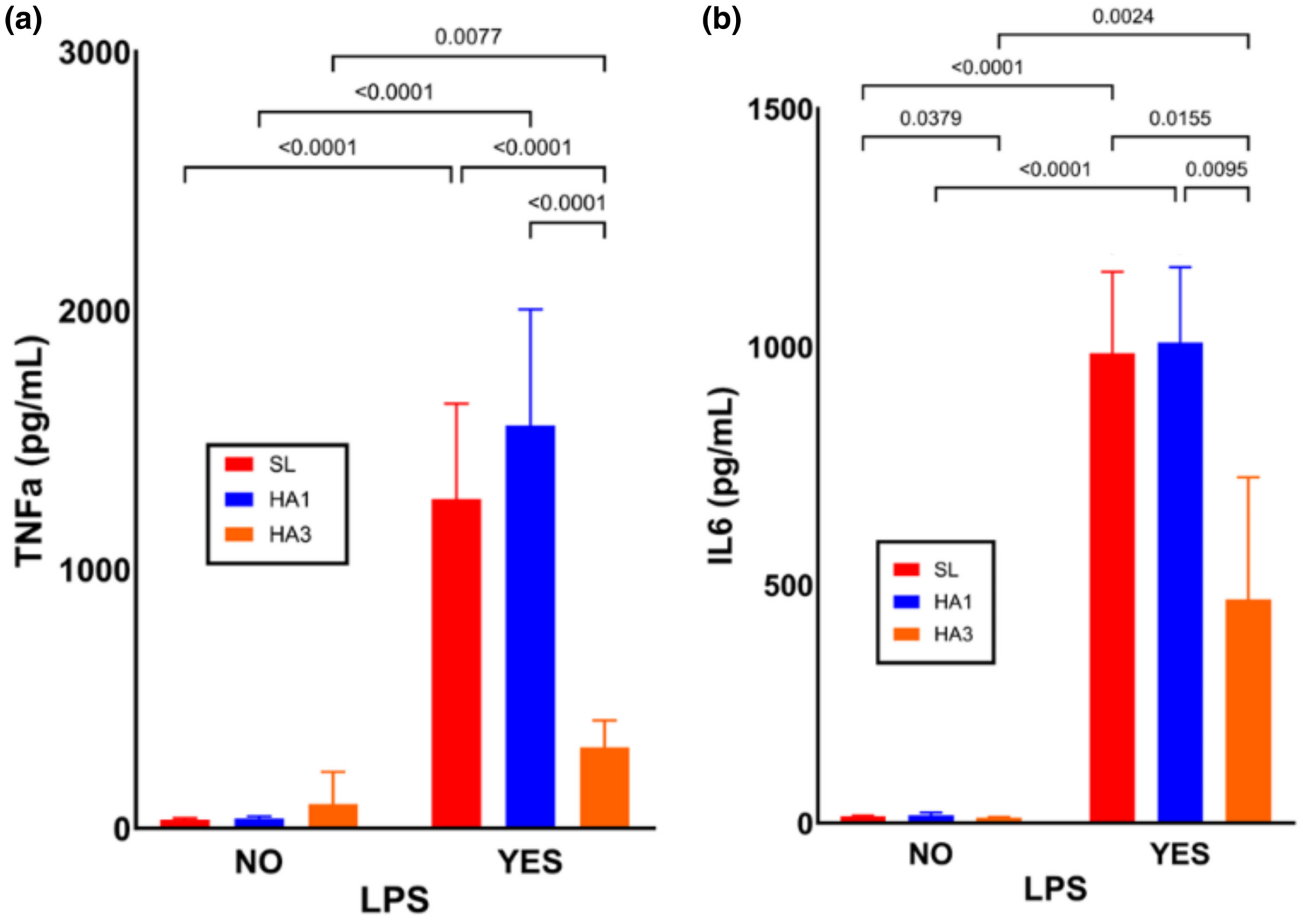
Inflammatory cytokine production in PBMCs collected at sea level and high altitude. PBMCs collected at sea level (SL), as well as after one (HA1) and three (HA3) days at high altitude were stimulated with LPS (100 ng/mL) for 6 h and analyzed for inflammatory cytokine production. Change in TNF‐α (a) and IL‐6 (b) production were quantified. Graphs are plotted as means with error bars representing 95% confidence intervals. Post‐hoc pairwise *t*‐test *p* values are provided for datasets showing significant main effects of location via two‐way repeated measures ANOVA.

PBMC cultures from cells collected in the same participants at sea level, as well as the first and third day at high altitude, were stimulated with LPS to determine if high‐altitude exposure modified immune cell reactivity to inflammatory stimuli. ELISA analysis on supernatants collected from each culture was performed to quantify TNF‐α (*n* = 17) and IL‐6 (*n* = 13) production. Overall, TNF‐α and IL‐6 production by immune cells after 1 day of high‐altitude exposure was not significantly changed compared to cells collected from the same individuals at sea level (*p* = 0.110 and *p* = 0.950, respectively) (Figure 4). In contrast, TNF‐α and IL‐6 production by PBMCs was significantly decreased in cells collected on day 3 at altitude compared to cells collected at sea level (*p* < 0.001 and *p* = 0.008, respectively) (Figure 4). Due to logistics during sample processing, PBMCs on day 3 at altitude were collected as buffy coat and subsequent isolation was completed after return to sea level, however in a separate experiment testing for impacts of these storage conditions, we found no significant impact on inflammatory responses or cell viability.

Of note, on the first day at altitude, 9 of the 17 participants demonstrated substantial increased TNF‐α production, despite this not being statistically significant overall (Figure S4). When participants were grouped based on if HA1 pro‐inflammatory cytokine production was greater than SL, there was no significant difference in AMS scores across these two groups. Lastly, there was no significant difference in TNF‐α or IL‐6 production by PBMCs on the first day at high altitude across AMS severity groups.

Further analyses for potential correlations between pro‐inflammatory cytokine production in response to LPS and AMS scores at altitude revealed no significant correlation between AMS score on day 1 and TNF‐α or IL‐6 production by PBMCs collected on day 1 during stimulation with LPS (TNF‐α: *r* = −0.14, *p* = 0.59; IL‐6: *r* = 0.21, *p* = 0.49) or day 3 at altitude (TNF‐α: *r* = 0.29, *p* = 0.28; IL‐6: *r* = 0.053, *p* = 0.87). Additionally, we also analyzed correlations with oxygen saturation (SpO_2_) and pro‐inflammatory cytokine production on the first day at high altitude, as systemic hypoxemia may impact immune cell function. There were no significant correlations between SpO_2_ with TNF nor IL‐6 production on first (TNF‐α: *r* = −0.075, *p* = 0.78; IL‐6: *r* = −0.042, *p* = 0.90) or third day (TNF‐α: *r* = −0.23, *p* = 0.39; IL‐6: *r* = −0.29, *p* = 0.35) at altitude.

### 3.4 Links between immune cell populations and Acute Mountain sickness

**FIGURE 5:**
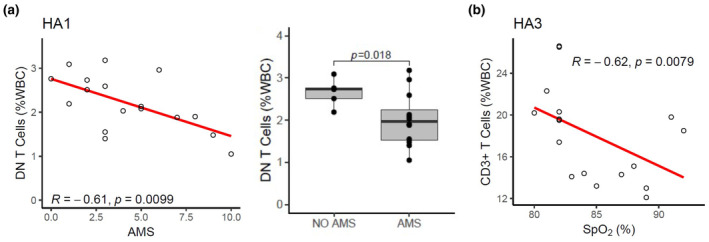
Significant correlations between immune cell populations and phenotypes at high altitude. Significant relationships were found between (a) double negative T cell populations and AMS scores on day 1 at altitude, as well as (b) CD3^+^ T cell populations and SpO_2_ on day 3 at altitude. *R* and *p* values for Pearson correlations are provided.

Immune cell populations (i.e., monocyte subsets, T cell subsets, B cells, NK cells) on the first and third day at altitude were tested for associations with self‐reported AMS scores and oxygen saturation (SpO_2_) collected at the same timepoint. Since only one participant scored ‘Severe AMS’, we combined the ‘Moderate’ and ‘Severe’ AMS groups. Of all analyzed associations with AMS scores and SpO_2_, the following were found to be significant: double negative T cells were negatively correlated with AMS score on the first day of altitude (*p* = 0.010, *r* = −0.61) (Figure 5a), CD3^+^ T Cells were negatively correlated with SpO_2_ on the third day at altitude (*p* = 0.008, *r* = −0.62) (Figure 5b). A complete description of all correlation analyses results is provided in Table S3 and Figure S5.

When analyzing total monocyte frequency and subset populations on both day 1 and 3 at altitude as a function of AMS severity groups (none, mild, or moderate–severe), we found that participants with moderate to severe AMS displayed more total monocytes compared to the mild AMS severity group (*p* = 0.009), but no difference from the group with no AMS. The moderate to severe AMS group also had increased classical monocytes compared to the mild group (*p* = 0.012), but no difference from the no AMS group. All analyses with immune populations and AMS severity groups (none, mild, or moderate–severe) are shown in Table S3 and Figure S6. We then further classified AMS groups into AMS+ versus AMS‐ on day 1 at high altitude based on a score of 3 or higher plus headache as an indicator of AMS. This analysis revealed significantly lower DN T Cells in the AMS group compared to the non‐AMS group (*p* = 0.018, Figure 5a, Figure S7). There were no significant relationships between immune cell populations and AMS groups on day 3 at high altitude.

**FIGURE 6:**
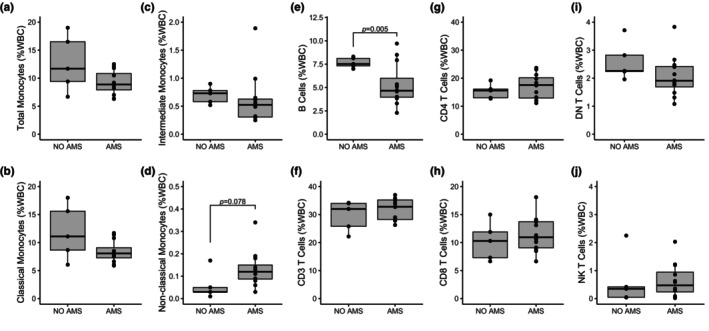
Differences in sea level baseline cell populations across AMS groups on the first day at high altitude. Panels represent comparisons of individual cell subpopulations across AMS groups, including (a) total monocytes, (b) classical monocytes, (c) intermediate monocytes, (d) non‐classical monocytes, (e) B cells, (f) CD3^+^ T Cells, (g) CD4^+^ T Cells, (h) CD8^+^ T Cells, (i) double negative (DN) T Cells, (j) and naturak killer (NK) T cells as a fraction of all white blood cells. Upper and lower box limits correspond to the first and third quartiles, thick center lines represent medians, and outliers outside 1.5 x IQR are represented as unconnected points. *p* Values are provided for groups showing significant or trending significant differences in immune cell populations across groups via unpaired *t*‐tests.

To determine if baseline immune cell populations affected AMS symptom severity at altitude, we analyzed associations between sea level immune populations with AMS scores on day 1 at altitude. Participants with higher baseline B cell levels showed a trend to be more likely to report no AMS compared to the mild and moderate–severe AMS groups (Figure S8, main effect of location: *F*(2, 22.6) = 11.3, *p* = 0.081). When separating AMS groups by AMS+ and AMS‐, this relationship was significant and individuals with lower B cells reported AMS while higher B cells at sea level may have indicated protection against AMS (Figure 6, *t*(13.6) = 3.37, *p* = 0.005). Furthermore, there was a strong trend that higher non‐classical monocytes at sea level were found in participants who had AMS on day 1 at high altitude (*t*(9.48) = −2.0, *p* = 0.078). This was coupled to trends towards a reduction in classical and intermediate monocytes in the AMS group.

## 4 DISCUSSION

In this study, we investigated how 3 days of high‐altitude acclimatization impacts immune cell balance in healthy sea‐level residents. We previously demonstrated that acute high‐altitude exposure triggers significant changes in inflammation‐related gene expression (Pham et al., 2022). In particular, we noted significant increases in the expression of genes encoding alarmins and damage associated molecular patterns (DAMPs), as well as many TLR4 pathway factors. However, how this acute inflammatory phenotype impacts immune cell development, differentiation, and activity remained unknown. In this subsequent study, we identified shifts in leukocyte populations, including monocyte subsets, B cells, and T cells throughout 3 days of high‐altitude acclimatization. In addition, we previously identified a potential hypoxia‐induced innate immune system sensitization mechanism through upregulation of TLR4 signaling pathway components at the gene expression level (Pham et al., 2022). Now, we specifically quantified TLR4 expression on the surface of immune cells and confirm that acute high‐altitude exposure does indeed promote greater TLR4 expression.

### 4.1 Acute high‐altitude exposure promotes a pro‐inflammatory innate immune phenotype

#### 4.1.1 Monocyte populations

Monocytes are classified as CD14^+^ CD16^+^. Specifically, monocyte subsets have been defined as classical (CD14^+^ CD16^−^), intermediate (CD14^+^ CD16^+^) and non‐classical (CD14^dim^ CD16^+^) (Marimuthu et al., 2018). Monocytes are essential innate immune cells that shape the immune response through their role in tissue healing, pathogen clearance, and activation of the adaptive immune system. Under periods of homeostasis, each monocyte subset is maintained in peripheral blood as they differentiate from classical to non‐classical phenotypes, constantly monitoring and being recruited to tissues to replenish tissue macrophages (Yang et al., 2014). In response to inflammation, monocytes are rapidly mobilized and recruited to sites of injury (Ingersoll et al., 2011; Serbina et al., 2003).

As these cells develop from the bone marrow, circulate throughout the blood, and extravasate into tissues, they experience a wide range of oxygen tensions. Sites of inflammation are commonly hypoxic, as the cellular demand for oxygen exceeds supply, and immune cells can experience oxygen tensions lower than 10 mmHg (Bosco et al., 2008; Fangradt et al., 2012; Sahaf et al., 2008; Strehl et al., 2014). While it is well known that hypoxic microenvironments impact immune cell properties, such as cytokine production and surface marker expression (Cramer et al., 2003), we found that acute systemic hypoxemia, or other factors related to acute high‐altitude exposure, also alter monocyte subsets.

In the present in vivo study, we found that monocyte subsets significantly favor the classical phenotype on the first day of high‐altitude exposure but shifted towards the intermediate subset by day 3 (Figure 1b,c). These data support the theory that acute high‐altitude hypoxia produces a pro‐inflammatory immune phenotype which resolves with acclimatization. To complement this data, research in rats at moderate altitude (1655 m) also found evidence of increased monocytic count, as well as greater pro‐inflammatory cytokine production in response to lipopolysaccharide (LPS) at high altitude compared to measures made at sea level, indicating a pro‐inflammatory profile at high altitude (Nguyen et al., 2021). In further agreement with our study, previous research on high altitude residents also found elevated monocyte counts compared to a sea‐level population (Bhattacharya et al., 2021). Additionally, studies with native Tibetan populations who are well adapted to the high altitude environment reveal that this group demonstrates reduced total monocytes and inflammatory classical monocytes compared to non‐Tibetans high altitude residents (Bhattacharya et al., 2021). This phenotype is attributed to an adaptive gain‐of‐function variant in *EGNL1* which encodes for prolyl hydroxylase (PHD), a key enzyme crucial for modulating HIF expression. This suggests a diminished hypoxia‐mediated inflammatory response may be an adaptive response to lifelong high altitude exposure.

Intermediate monocyte subsets were significantly increased by day 3 of acclimatization (Figure 1c). This result may support other studies that find elevated intermediate monocytes in HAPH and HAPE patients compared to healthy controls at the same elevation (Bhattacharya et al., 2021; Wu et al., 2023). Although both studies reported that the classical monocyte subsets were not different between healthy controls and patients with HAPH or HAPE, they did find a significant increase in total monocyte count in peripheral whole blood, as well as an increase in both intermediate and non‐classical monocyte subsets in the HAPH and HAPE groups. Furthermore, Wu et al. report that intermediate monocyte populations were significantly increased in HAPH patients compared to healthy controls at the same elevation.

Overall, our data suggests that acute high‐altitude exposure initially promotes a pro‐inflammatory phenotype in monocytes that transitions to an anti‐inflammatory phenotype over the course of acclimatization. Monocyte subsets are suspected to return to baseline distributions, even if total monocyte count remains elevated, following acclimatization to high‐altitude. While our study spans a short timescale of acclimatization, another study with longer hypoxia exposure in non‐native sojourners also demonstrated immune sensitization to inflammatory stimuli, however total monocyte counts did not peak until 4–7 months at high altitude (Feuerecker et al., 2019). These findings warrant further investigation of the impact of hypoxia‐induced changes in immune cell phenotypes, as both inflammatory and non‐inflammatory monocyte subsets are suspected to play a role in hypoxia‐induced pathologies.

#### 4.1.2 Impact of acute high altitude hypoxia exposure on inflammatory sensitivity

In a previous study, we hypothesized that acute high‐altitude exposure causes increases responsiveness of immune cells to inflammatory stimuli due to upregulation of TLR4 signaling pathway genes (*CD14*, *LY96*, *TLR4*) and alarmins (Pham et al., 2022). When analyzing if immune cells at high altitude were more responsive to inflammatory stimuli, on average we did not find any significant elevation in pro‐inflammatory TNF‐a or IL‐6 cytokine production following LPS stimulation (Figure 4). However, on an individual basis, many participants showed an appreciable increase in TNF‐α production from PBMCs collected on the first day at altitude compared to sea level values (Figure S4). Interestingly, there was a significant reduction in pro‐inflammatory cytokine production on the third day at altitude (Figure 4). This could be an effect of immunosuppression, as HIF pathway activation in hypoxia also plays a major antioxidant role. Since none of our participants developed any pathology more severe than AMS, and only one participant experienced severe AMS, it is possible that in severe high‐altitude illnesses, immune sensitization may be more apparent. Altogether, this data highlights a possible mechanism of hypoxia‐induced immune sensitization and adaptation worthy of future investigation.

### 4.2 Adaptive immune cells are affected by acute high‐altitude exposure

B lymphocytes produce antibodies that mediate protection against pathogens. The impact of hypoxia on B cell function remains controversial (Zhang et al., 2022). There are conflicting reports regarding how hypoxia regulates B cell populations and their function at high altitude. Several studies report that high altitude does not alter B cell population or function (Facco et al., 2005; Meehan, 1987), while others report increases in both of these parameters (Chohan et al., 1975; Feuerecker et al., 2019; Mishra & Ganju, 2010). Our findings support the conclusion that acute high‐altitude exposure does increase B cell populations (Figure 1e). Studies have found that hypoxia favors generation of plasma cells, which are the endpoint of B cell differentiation (Schoenhals et al., 2017). This corresponds with work that shows increases in antibody production in response to high‐altitude (Chohan et al., 1975; Tengerdy & Kramer, 1968). However, long‐term chronic hypoxia exposure may have the opposite effect. Studies on high‐altitude native Tibetans and long‐term high‐altitude residents of Han Chinese ancestry reveal that total B cells were significantly reduced at altitude in both groups compared to a mid‐altitude Han Chinese population. (Bai et al., 2022). This may be an effect of the evolutionarily conserved response to hypoxic immunological niches and modulation of stage‐specific HIF expression throughout B cell development (Burrows et al., 2020; Zhang et al., 2022). The initial increase in B cells seen in our study may be an effect on B cells already differentiated, but chronic hypoxic exposure prevents the development of new immature B cells, leading to a reduction in circulating B cells in long‐term high‐altitude residents.

T lymphocyte balance has also been found to be affected by acute high‐altitude hypoxia exposure (Facco et al., 2005; Feuerecker et al., 2019; Klokker et al., 1993; Meehan et al., 1988; Mishra & Ganju, 2010). Likewise, our data show that total CD3^+^ T cells were significantly reduced throughout high‐altitude exposure, including both CD4^+^ T cells and CD8^+^ T cells (Figure 1f–h).

CD4^+^ T cells are critical in activating and modulating the adaptive immune response. While our study does not distinguish between specific CD4^+^ helper subsets (T‐helper cells 1 (Th1) vs T‐helper cells 2 (Th2)), previous research demonstrates that the Th1/Th2 immune balance was dysregulated at high altitude (Caldwell et al., 2001; Facco et al., 2005). Furthermore, studies have also found that T cells cultured in hypoxic conditions or collected at high altitude had significantly reduced function and proliferative response when stimulated with mitogen (PHA) (Facco et al., 2005; Klokker et al., 1993; Mishra & Ganju, 2010; Tingate et al., 1997). However, this is controversial, as conflicting studies have found no change or even increased function and cytokine production following stimulation (Caldwell et al., 2001; Feuerecker et al., 2019). Because hypoxia is a prominent feature in inflammation, cancer, or tumor microenvironments, studies have found that hypoxia inhibits CD4^+^ effector function through immunosuppression via regulatory T cells (Karger et al., 2017). Specifically, HIF‐1α stability and function was implicated in the exacerbation of the regulatory T cell suppressive capacity in hypoxia (Ben‐Shoshan et al., 2008; Lee et al., 2015). Therefore, the decrease in circulating CD4^+^ T cells may not be due to T cell exhaustion, but suppression of CD4^+^ population by regulatory T cells.

### 4.3 Immune cell populations and AMS correlations

While it remains to be determined if, or how, pre‐existing chronic inflammation may play a part in high altitude pathologies, we hypothesized that increased pro‐inflammatory immune cell phenotypes are a driving force for AMS. We found that baseline monocyte levels (total, classical, intermediate, non‐classical) did not significantly predict AMS severity on either the first or third day at altitude, but show a trend for higher AMS in participants with higher non‐classical monocytes at baseline (Figure 6a–d). This suggests that elevated non‐classical monocytes may be an early indicator of high‐altitude illness development, as participants with higher non‐classical monocytes had worse AMS severity on average. Non‐classical monocytes normally serve a protective and anti‐inflammatory role in maintaining vascular homeostasis; however, this monocyte subset also contributes to chronic inflammatory diseases. Indeed, non‐classical monocytes have been implicated in pathogenesis of inflammatory disease, such as rheumatoid arthritis and systemic lupus erythematosus (Narasimhan et al., 2019). Non‐classical monocytes are notably recruited to small pulmonary arteries and promote vascular remodeling, and the accumulation of non‐classical monocytes may contribute to pulmonary hypertension (Yu et al., 2020). Together, this suggests that in acute high‐altitude exposure, non‐classical monocytes are actively recruited into pulmonary vasculature, and elevated non‐classical monocyte concentration may lead to excessive monocyte‐endothelial cell interaction (Liang et al., 2019). While we suspect that an anti‐inflammatory phenotype is beneficial to acclimatization, the direct infiltration of non‐classical monocytes may play a direct role in the initiation or exacerbation of high‐altitude illness pathogenesis.

Finally, participants with no AMS had a higher baseline B cells compared to AMS groups (Figure 6e). This could potentially indicate a protective role of B cells on an acute hypoxia exposure scale. While there are multiple publications that report both enhanced as well as suppressed antibody production in animal and human models at altitude (Chohan et al., 1975; Meehan, 1987; Singh et al., 1977; Tengerdy & Kramer, 1968), this may be indicative of the benefit of functional antibody production. Recently, research on the severe acute respiratory syndrome coronavirus‐2 (SARS‐CoV‐2) at altitude has found elevated and sustained humoral immune response (Tomas‐Grau et al., 2021). This suggests that participants with higher baseline B cells may have a more protective phenotype against the development of high‐altitude illness, as well as protection against exacerbated response to subsequent infections at altitude. Further research is necessary to elucidate antibody production and function at high altitude, and if these factors promote or suppress high‐altitude pathology development and/or exacerbation.

## 5 LIMITATIONS

One limitation of our study is our moderate sample size (*n* = 20), although the paired design allowed for stronger detection of changes in immune populations, as sea level samples served as a paired control for high altitude samples in the same individual. Additionally, our study group included both men and women, however we did not focus on potential sex‐specific changes in immune population or function. Future experimental designs exploring potential differences in the impact of high altitude on immune function on the basis of sex will be essential. Furthermore, only one participant developed severe AMS after acute high‐altitude exposure, and therefore we could not identify significant associations between severe AMS and specific changes in immune function or population distributions. A larger sample size with a wider range of AMS severity will provide stronger power to identify potential associations. This study also did not measure absolute white blood cell count and focuses only on changes in immune phenotype, as well as immune cell reactivity. These findings are significant because they provide insight into how shifts in immune cell populations may contribute to high‐altitude pathologies and exacerbate inflammatory responses to subsequent stimuli. However, future work will identify absolute changes in immune cell numbers in addition to cell phenotype shifts. Finally, this study cannot tease apart the independent effects of high‐altitude stressors such as hypobaric pressure, hypoxia, and low temperatures on these immune outcomes.

## 6 CONCLUSION

In conclusion, we demonstrate that acute high‐altitude exposure significantly alters both innate and adaptive immune cell populations. Specifically, our analysis reveals evidence of innate immune sensitization, most notably in monocyte subsets, as well as adaptive immune suppression, particularly CD4^+^ T cells. While our data suggests acute high‐altitude exposure initially promotes a pro‐inflammatory phenotype that transitions to an anti‐inflammatory phenotype over the course of acclimatization, further studies are necessary to elucidate underlying mechanisms behind hypoxia‐induced inflammation and its contribution to high‐altitude illnesses. In particular, comparing high‐altitude native populations displaying adaptive and maladaptive phenotypes in this environment might expand our understanding of the time domains of hypoxia‐induced inflammation. Characterizing immune responses to inflammatory stimuli at high altitude would also provide mechanistic insight into hypoxia‐induced immune sensitization.

## AUTHOR CONTRIBUTIONS

KP and ECH conceived and designed the research. KP, SF, and ECH assisted in sample collection. KP, SS, and ECH analyzed data, interpreted the results of experiments, and prepared figures. KP drafted the manuscript. KP and ECH edited and revised the manuscript. All authors approved of this manuscript.

## CONFLICT OF INTEREST STATEMENT

The authors declare that the research was conducted in the absence of any commercial or financial relationships that could be construed as a potential conflict of interest.

## ETHICS STAETMENT

This study was approved by the University of California, Riverside Clinical Institutional Review Board (HS 22‐088). All participants were informed of the study's purpose and risks. Participants provided written informed consent in their native language (English). The work was conducted in accordance with the Declaration of Helsinki, except for registration in a database.

## Supporting information


**Figure S1.** Full gating strategy for identifying PBMC population subsets. A representative sea‐level baseline sample is illustrated for immune characterization flow gating strategy.

**Figure S2.** Full gating strategy for CD14^+^ TLR4+ high altitude PBMCs. Representative sea‐level baseline sample example for TLR4 flow gating control.

**Figure S3.** Monocyte subset immune population analysis during 3 days of acute high‐altitude exposure. Quantification of monocyte subpopulations from total monocyte (%). Graphs are plotted as mean and error bars as 95% confidence interval.

**Figure S4.** Inflammatory cytokine production in PBMCs collected at sea level and high altitude. PBMCs collected at sea level (SL), as well as after one (HA1) and three (HA3) days at high altitude were stimulated with LPS (100 ng/mL) for 6 h and analyzed for inflammatory cytokine production. Changes in TNF‐α (A) and IL‐6 (B) production were quantified. Lines connect data from the same participant. Post‐hoc pairwise *t*‐test *p* values are provided for datasets showing significant main effects of location via two‐way repeated measures ANOVA.

**Figure S5.** Correlation matrix for all cell populations, AMS, and pulse oxygen saturation (SpO_2_). The left plot provides correlations across variables on the first day at high altitude (HA1) and the right plot provides correlations across variables on the third day at high altitude (HA3). Boxes with circles indicate the presence of a significant correlation with *p* < 0.05. Empty boxes indicate no significant correlation. Circle colors represent Pearson correlation coefficients.

**Figure S6.** Relationships between immune cell populations and AMS severity on the first day at high altitude. Upper and lower box limits correspond to the first and third quartiles, thick center lines represent medians, and outliers outside 1.5 * IQR are represented as unconnected points. Post‐hoc pairwise *t*‐test *p* values are provided for groups showing significant main effects of location via one‐way ANOVA. AMS severity groups: None (0–2), Mild (3–5). Moderate–Severe (6+).

**Figure S7.** Relationships between immune cell populations and AMS severity on the first day at high altitude when grouped by AMS+ and AMS‐. Upper and lower box limits correspond to the first and third quartiles, thick center lines represent medians, and outliers outside 1.5 * IQR are represented as unconnected points. P values are provided for groups showing significant differences via unpaired *t*‐tests. AMS severity groups: No AMS (0–2), AMS (3+).

**Figure S8.** Relationships between sea level baseline immune cell populations and AMS severity on the first day at high altitude. Upper and lower box limits correspond to the first and third quartiles, thick center lines represent medians, and outliers outside 1.5 * IQR are represented as unconnected points. Post‐hoc pairwise *t*‐test *p* values are provided for groups showing significant main effects of location via one‐way ANOVA. AMS severity groups: None (0–2), Mild (3–5). Moderate–Severe (6+).

**Table S1.** Flow Cytometry Immune Cell Characterization Panel.

**Table S2.** Flow Cytometry TLR4 Surface Expression Panel.

**Table S3.** Pearson correlation analyses of all immune cell populations and AMS scores and oxygen saturation (SpO2) on day 1 and 3 at altitude.

## Data Availability

All data reported here will be made publicly available at the time of publication.
