# Supplementary figures and images for: Changes in immune cell populations during acclimatization to high altitude
Source: Physiol Rep. 2024 Nov 17;12(22):e70024. doi: 10.14814/phy2.70024 (PMC11570420; doi:10.14814/phy2.70024)

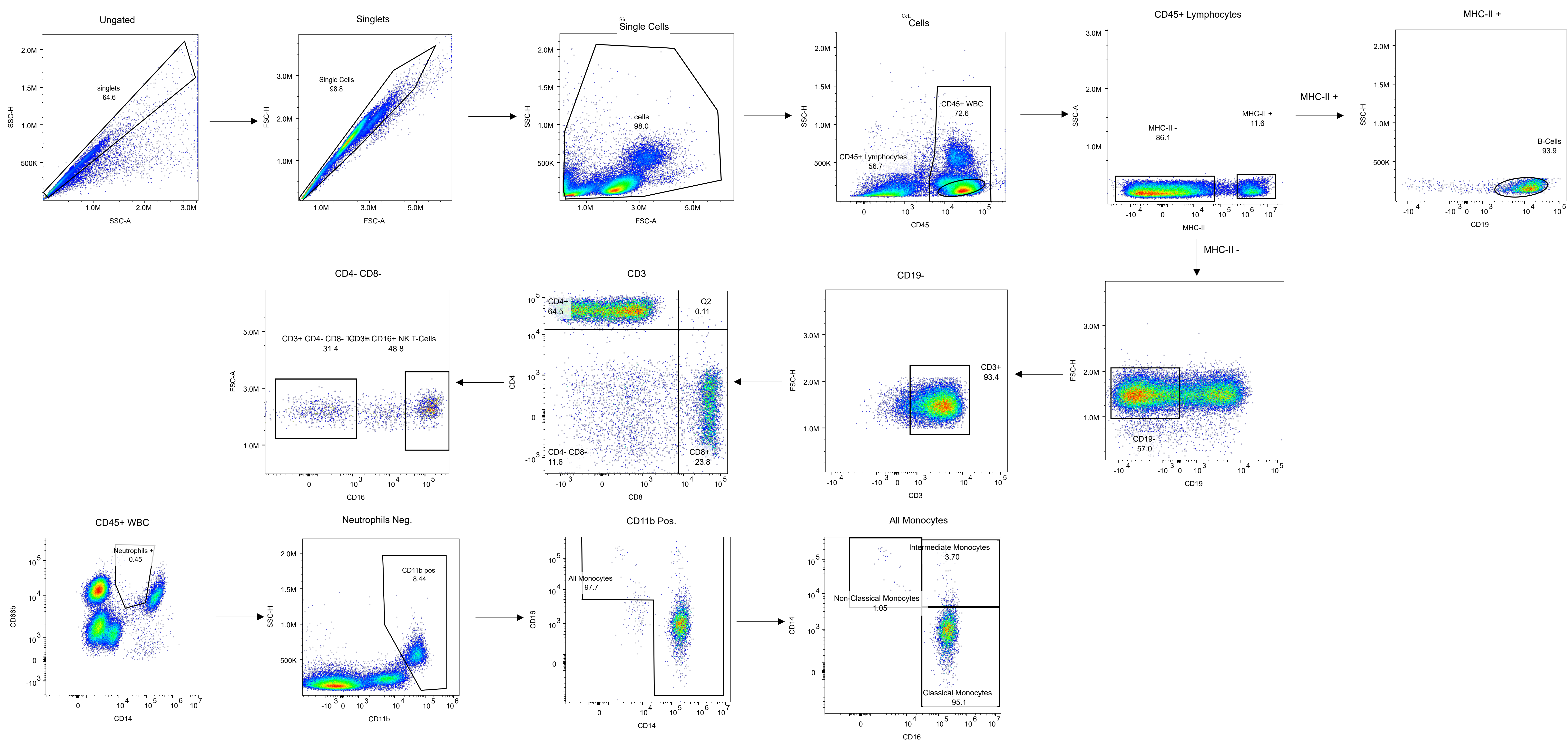

Supplement: Supplementary file 1 — Figure S1. Full gating strategy for identifying PBMC population subsets. A representative sea‐level baseline sample is illustrated for immune characterization flow gating strategy. [file PHY2-12-e70024-s004.pdf]

Ungated

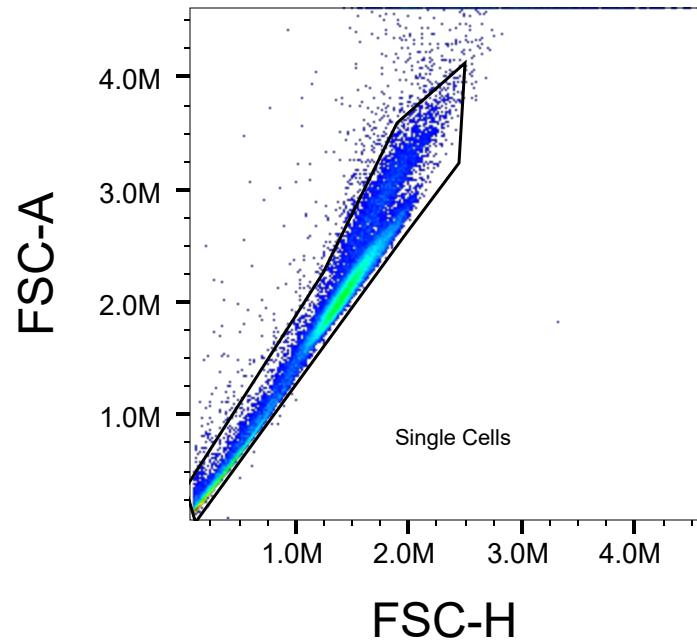

Live / Dead Stain

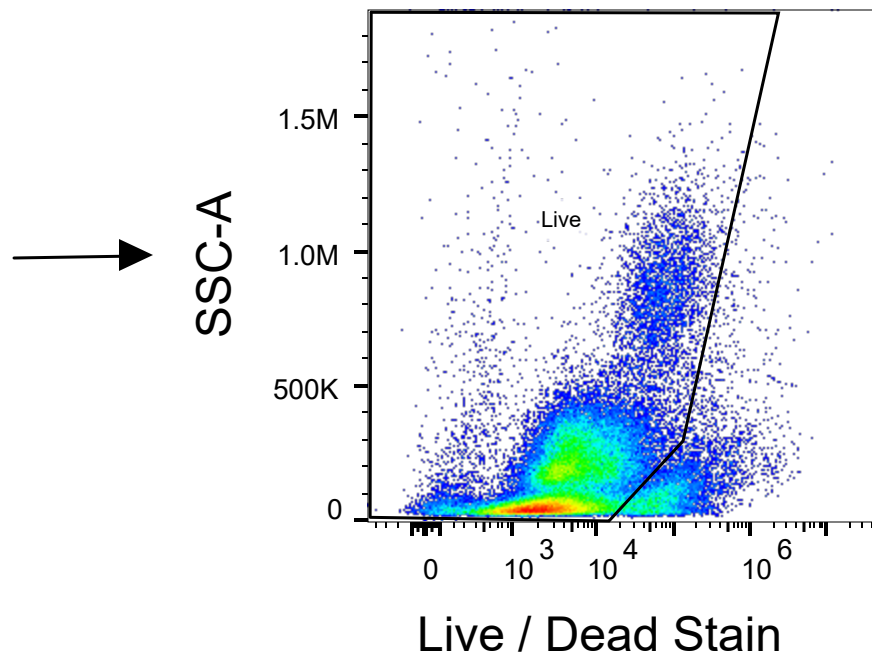

CD14+ TLR4+

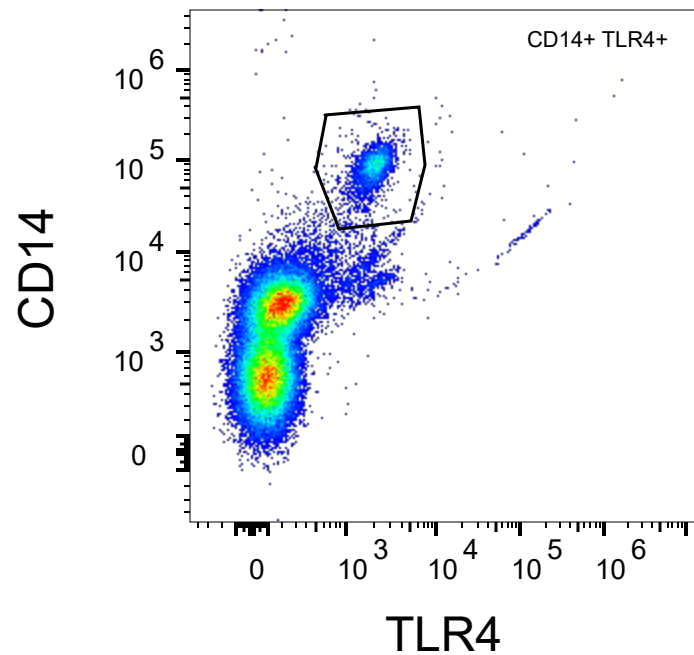

Supplement: Supplementary file 2 — Figure S2. Full gating strategy for CD14+ TLR4+ high altitude PBMCs. Representative sea‐level baseline sample example for TLR4 flow gating control. [file PHY2-12-e70024-s002.pdf]

### Classical M (% Monocytes)

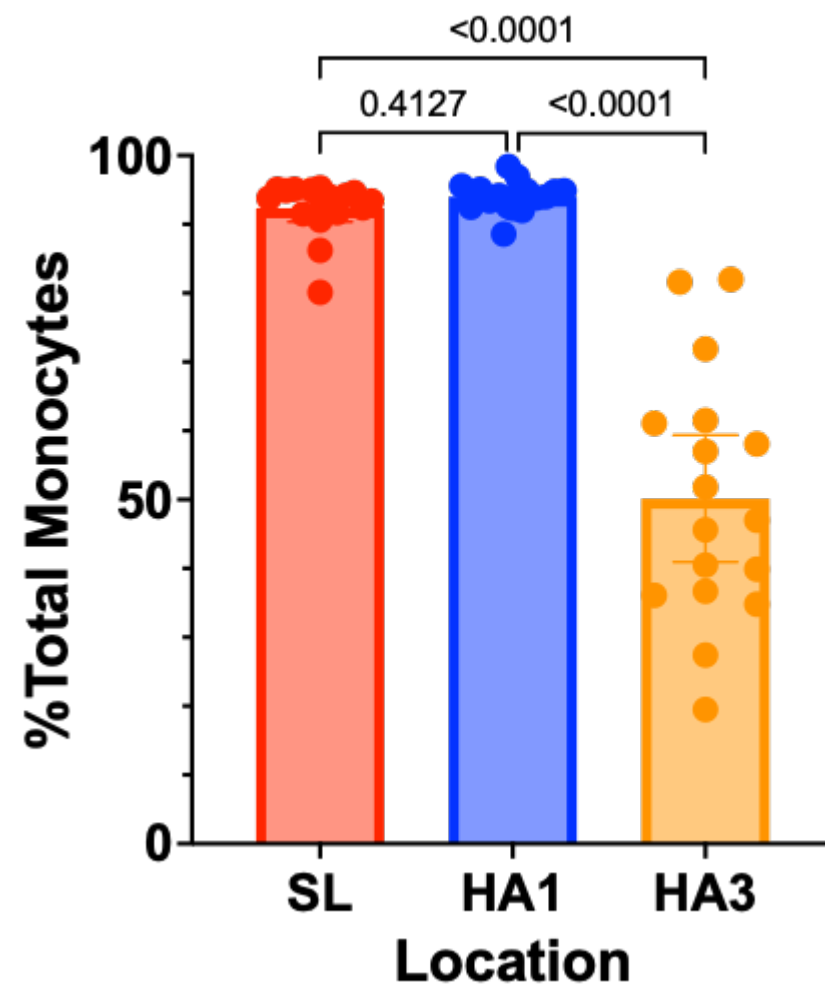

### Intermediate M (% Monocytes)

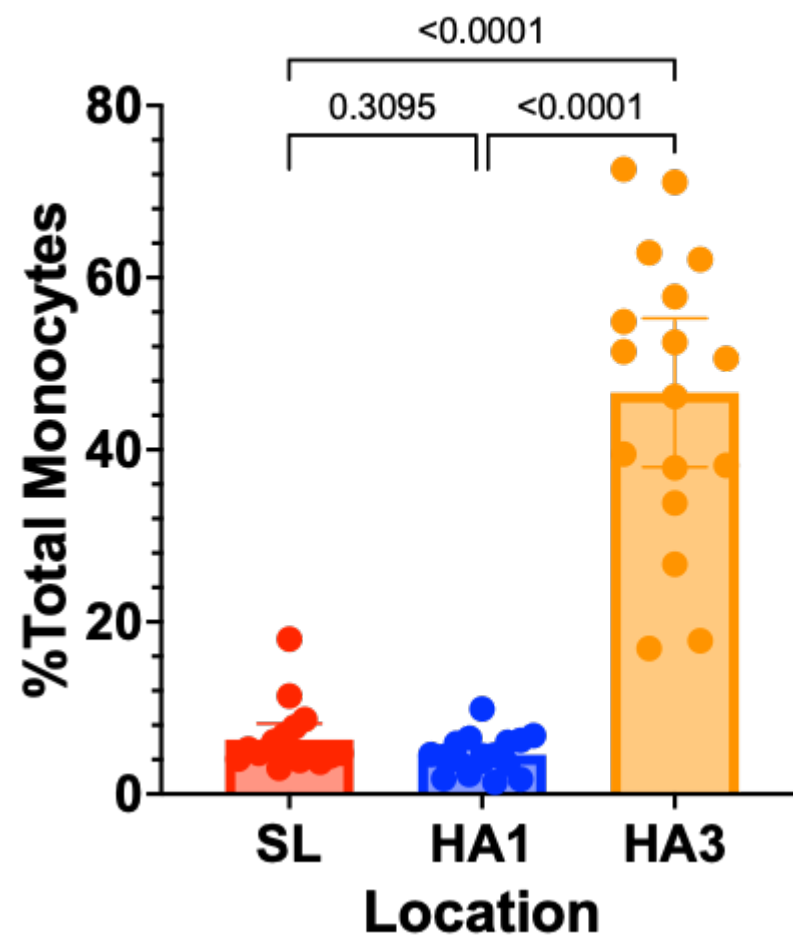

### Non-Classical Monocytes (%M)

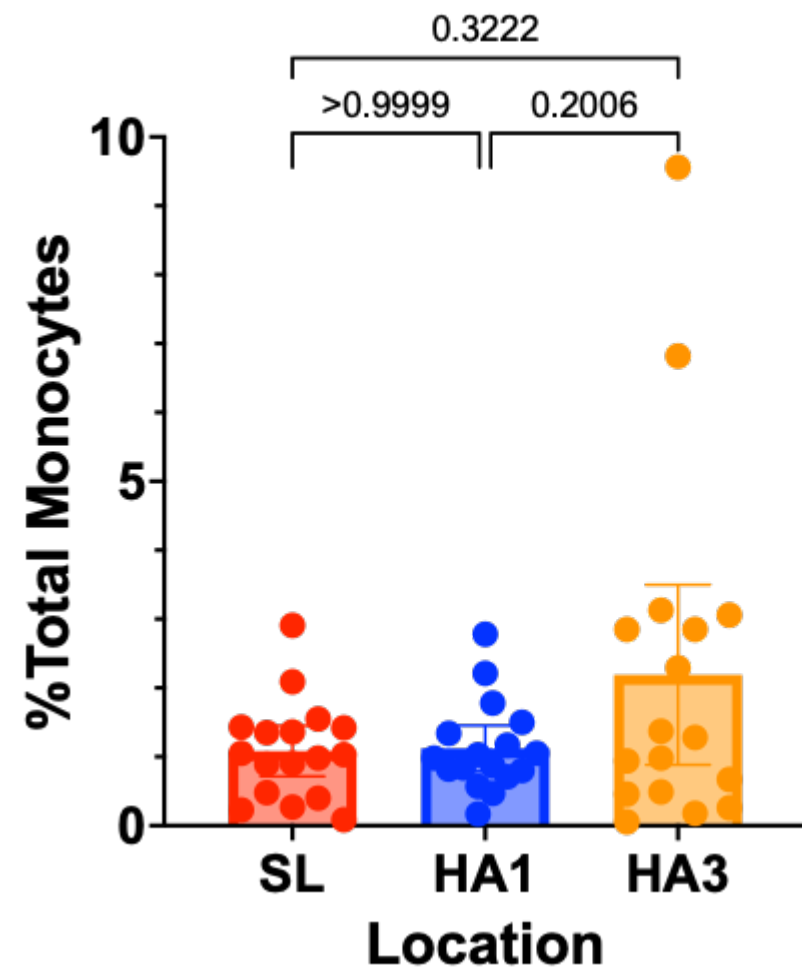

Supplement: Supplementary file 3 — Figure S3. Monocyte subset immune population analysis during 3 days of acute high‐altitude exposure. Quantification of monocyte subpopulations from total monocyte (%). Graphs are plotted as mean and error bars as 95% confidence interval. [file PHY2-12-e70024-s010.pdf]

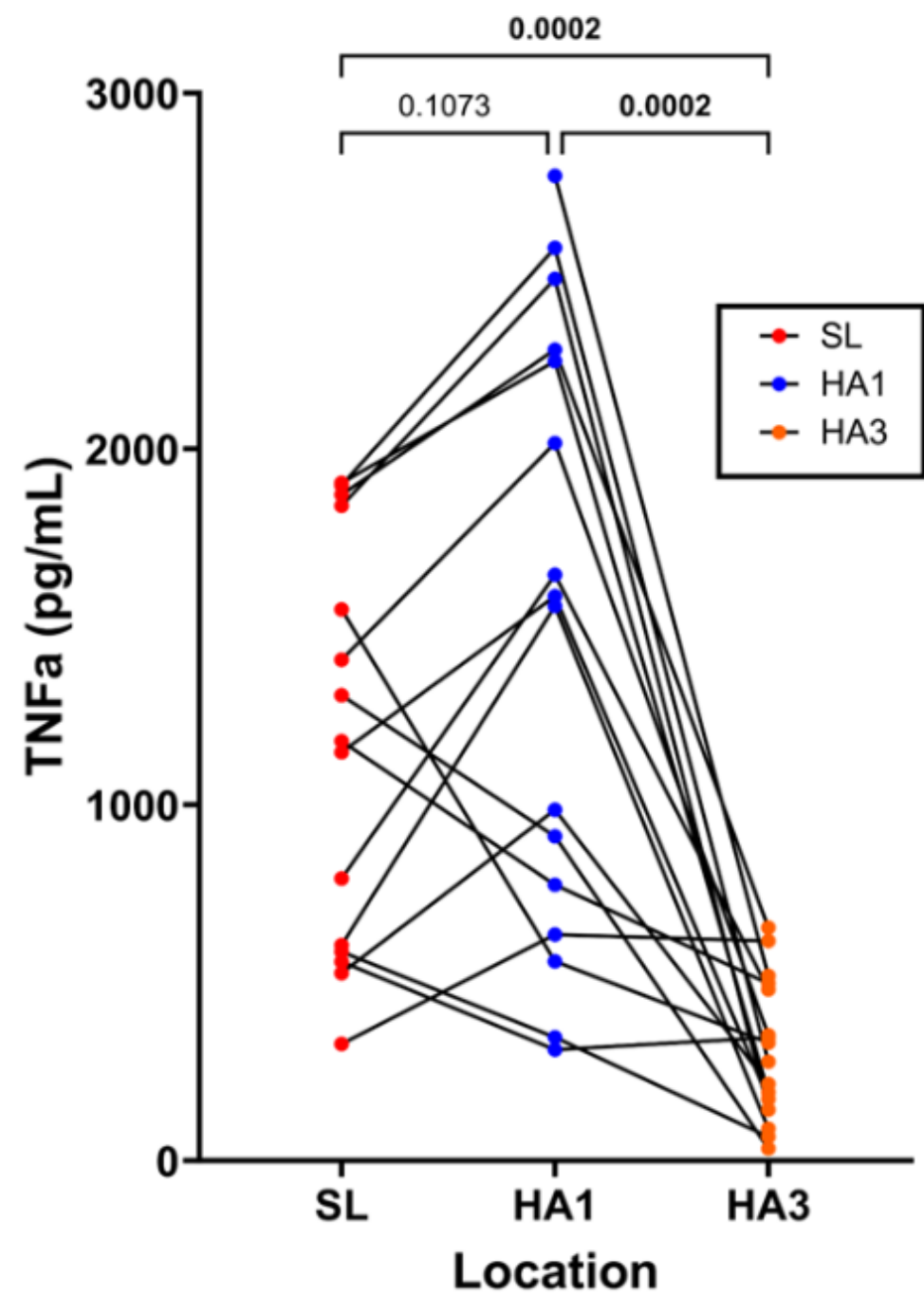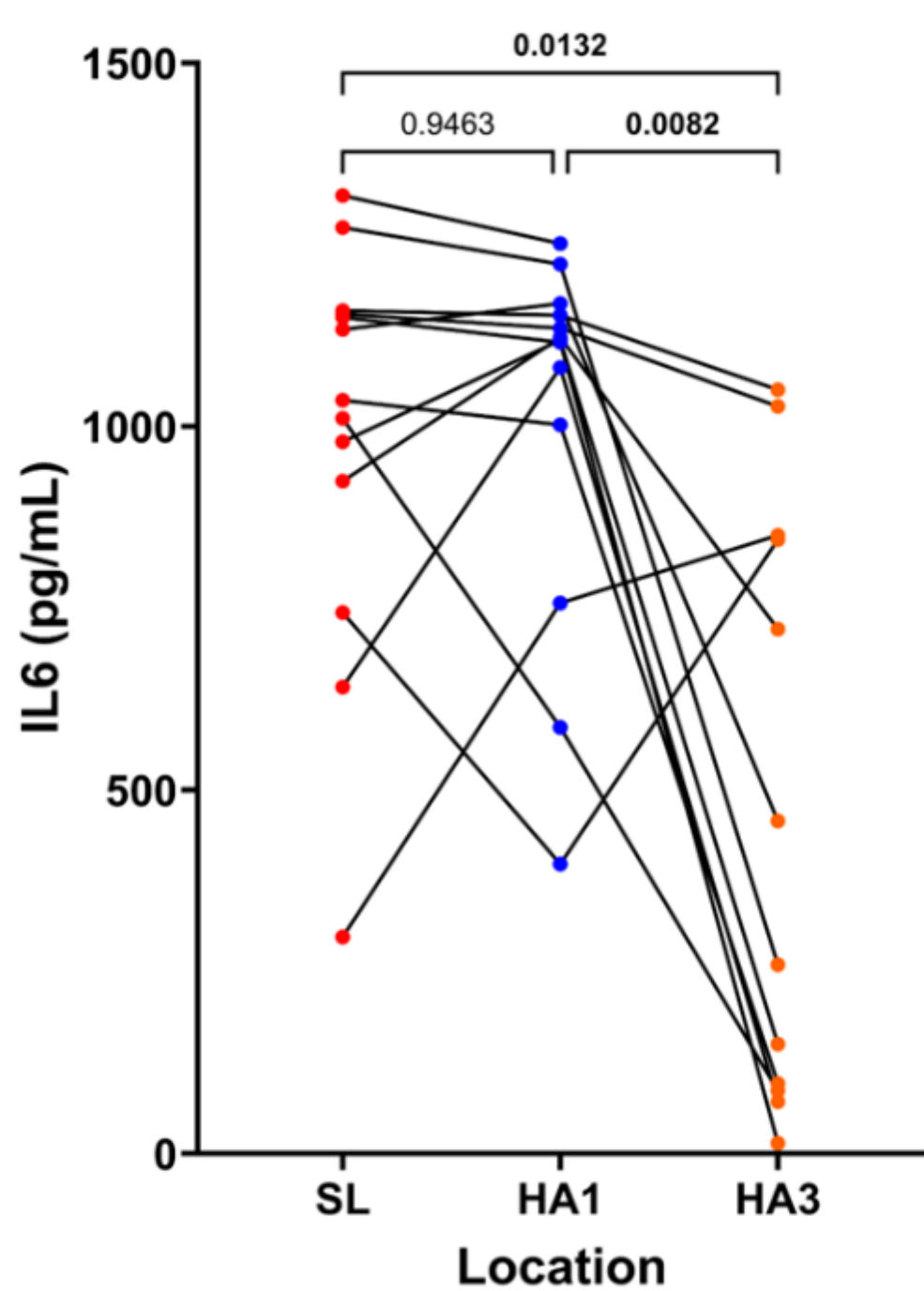

Supplement: Supplementary file 4 — Figure S4. Inflammatory cytokine production in PBMCs collected at sea level and high altitude. PBMCs collected at sea level (SL), as well as after one (HA1) and three (HA3) days at high altitude were stimulated with LPS (100 ng/mL) for 6 h and analyzed for inflammatory cytokine production. Changes in TNF‐α (A) and IL‐6 (B) production were quantified. Lines connect data from the same participant. Post‐hoc pairwise t‐test p values are provided for datasets showing significant main effects of location via two‐way repeated measures ANOVA. [file PHY2-12-e70024-s006.pdf]

# HA1

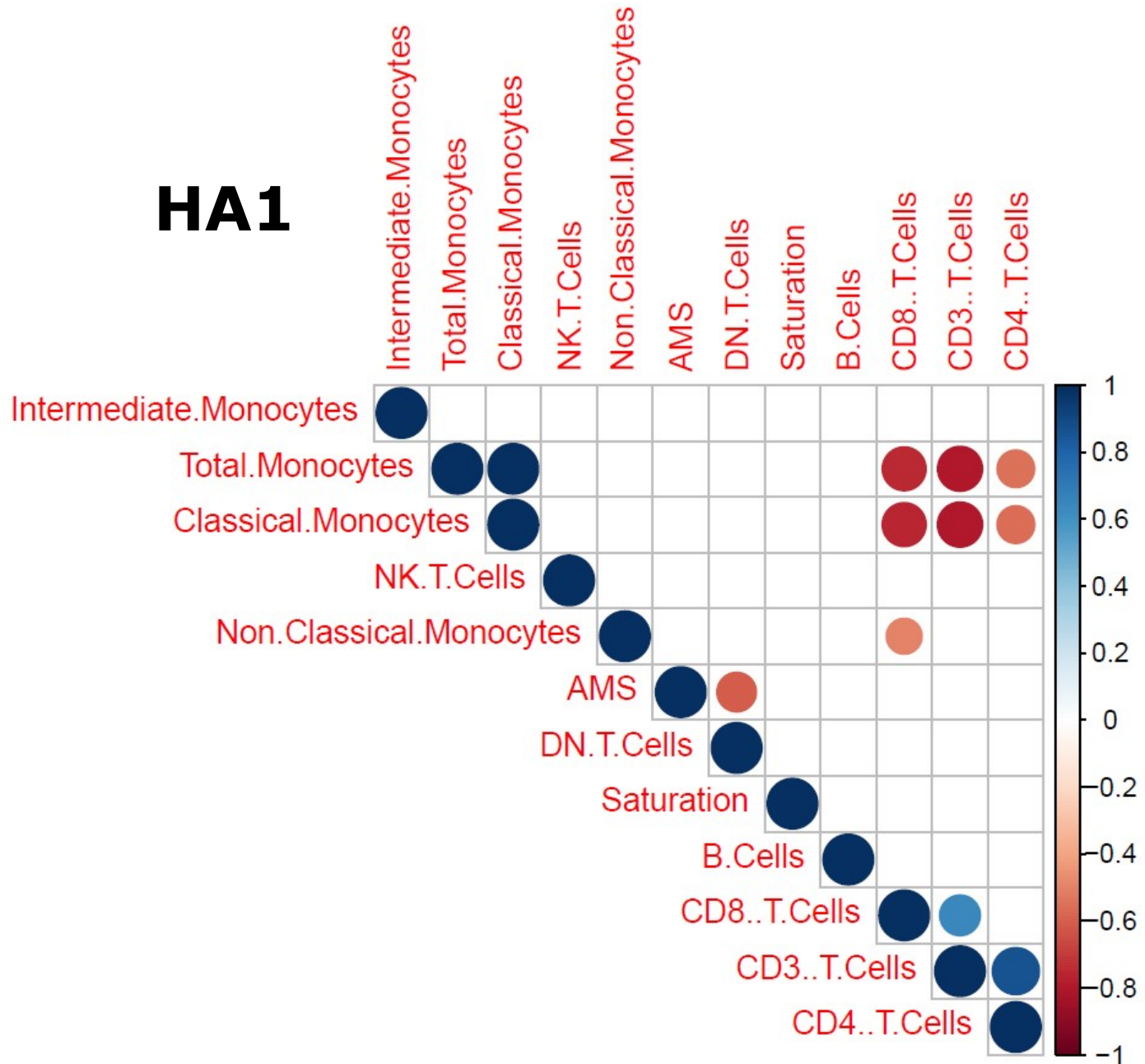

# HA3

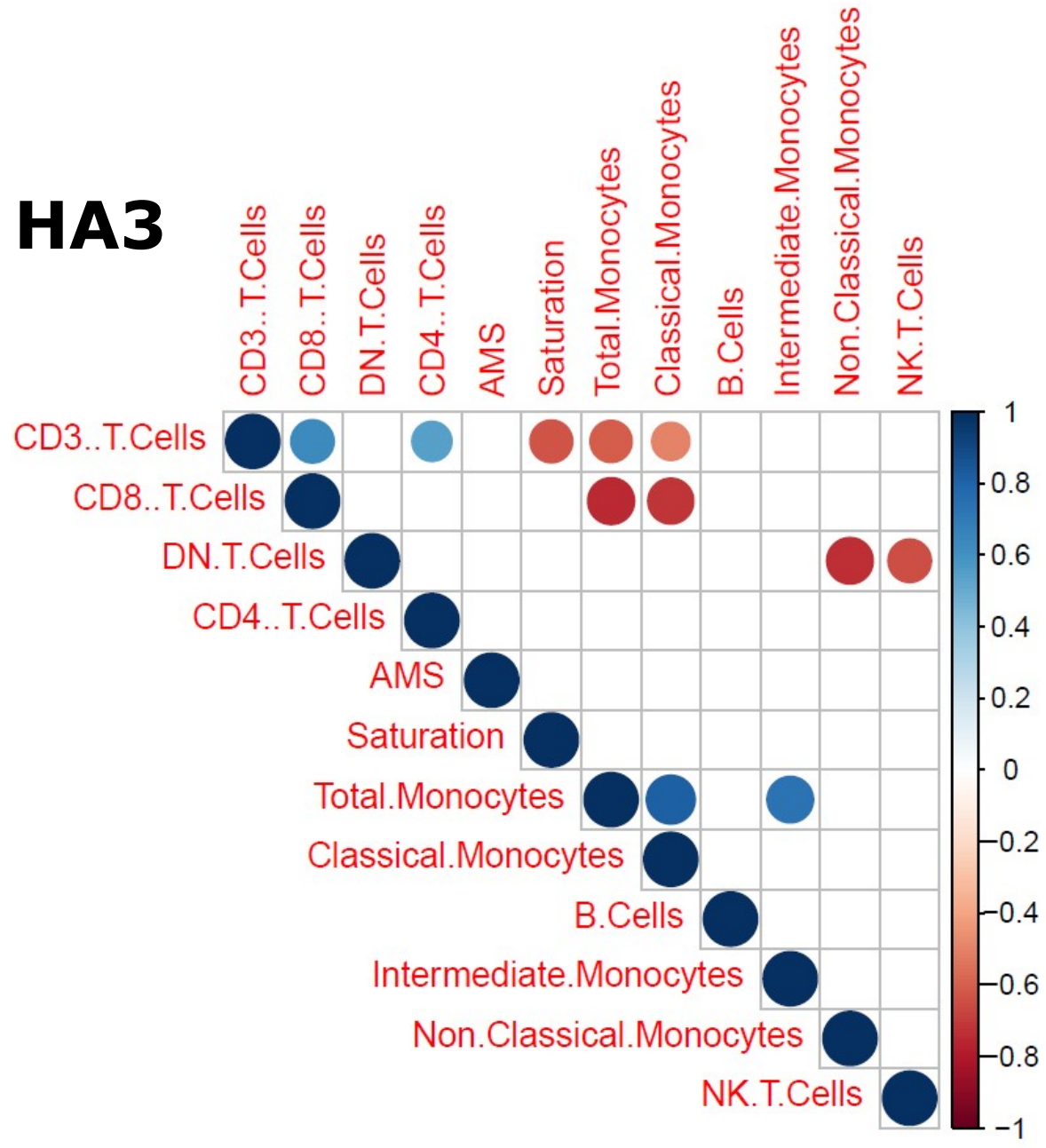

Supplement: Supplementary file 5 — Figure S5. Correlation matrix for all cell populations, AMS, and pulse oxygen saturation (SpO2). The left plot provides correlations across variables on the first day at high altitude (HA1) and the right plot provides correlations across variables on the third day at high altitude (HA3). Boxes with circles indicate the presence of a significant correlation with p < 0.05. Empty boxes indicate no significant correlation. Circle colors represent Pearson correlation coefficients. [file PHY2-12-e70024-s003.pdf]

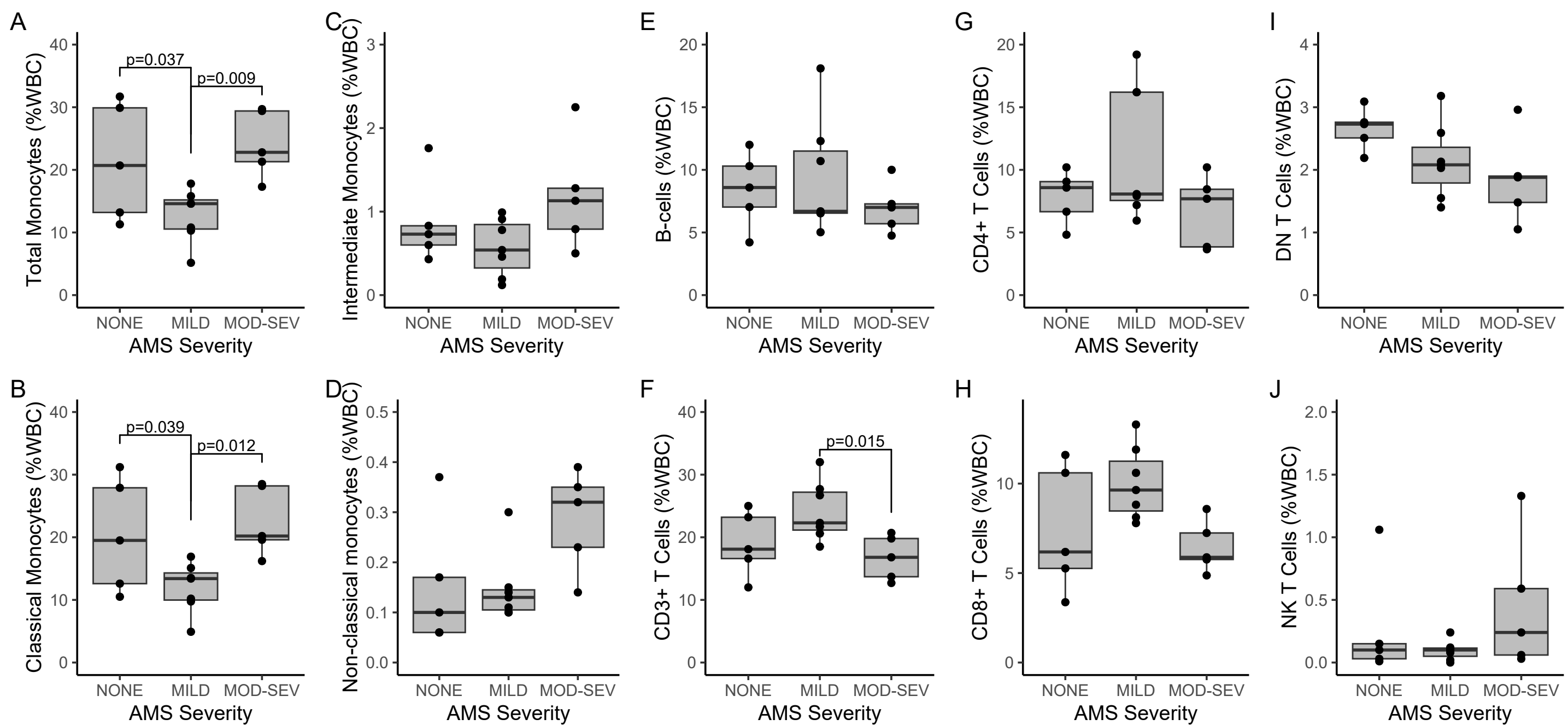

Supplement: Supplementary file 6 — Figure S6. Relationships between immune cell populations and AMS severity on the first day at high altitude. Upper and lower box limits correspond to the first and third quartiles, thick center lines represent medians, and outliers outside 1.5 * IQR are represented as unconnected points. Post‐hoc pairwise t‐test p values are provided for groups showing significant main effects of location via one‐way ANOVA. AMS severity groups: None (0–2), Mild (3–5). Moderate–Severe (6+). [file PHY2-12-e70024-s005.pdf]

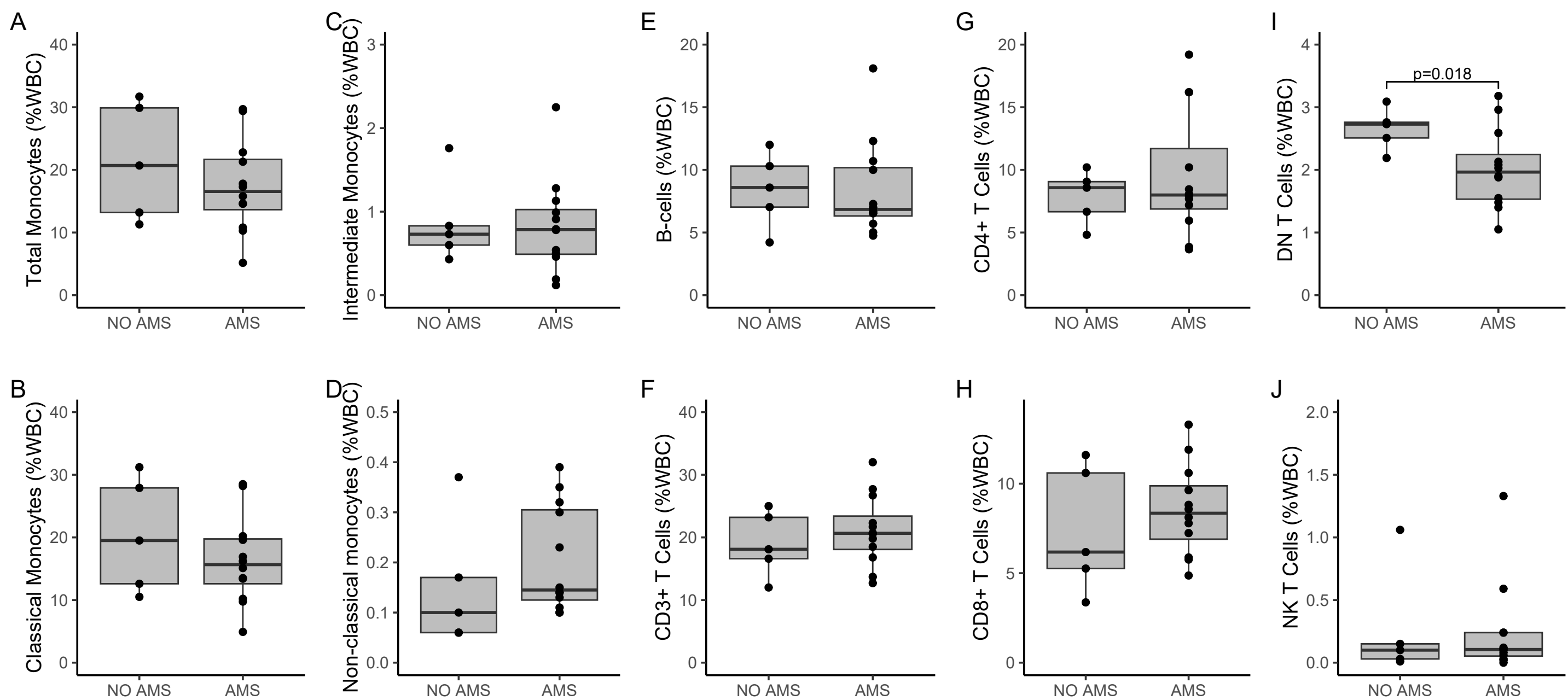

Supplement: Supplementary file 7 — Figure S7. Relationships between immune cell populations and AMS severity on the first day at high altitude when grouped by AMS+ and AMS‐. Upper and lower box limits correspond to the first and third quartiles, thick center lines represent medians, and outliers outside 1.5 * IQR are represented as unconnected points. P values are provided for groups showing significant differences via unpaired t‐tests. AMS severity groups: No AMS (0–2), AMS (3+). [file PHY2-12-e70024-s001.pdf]

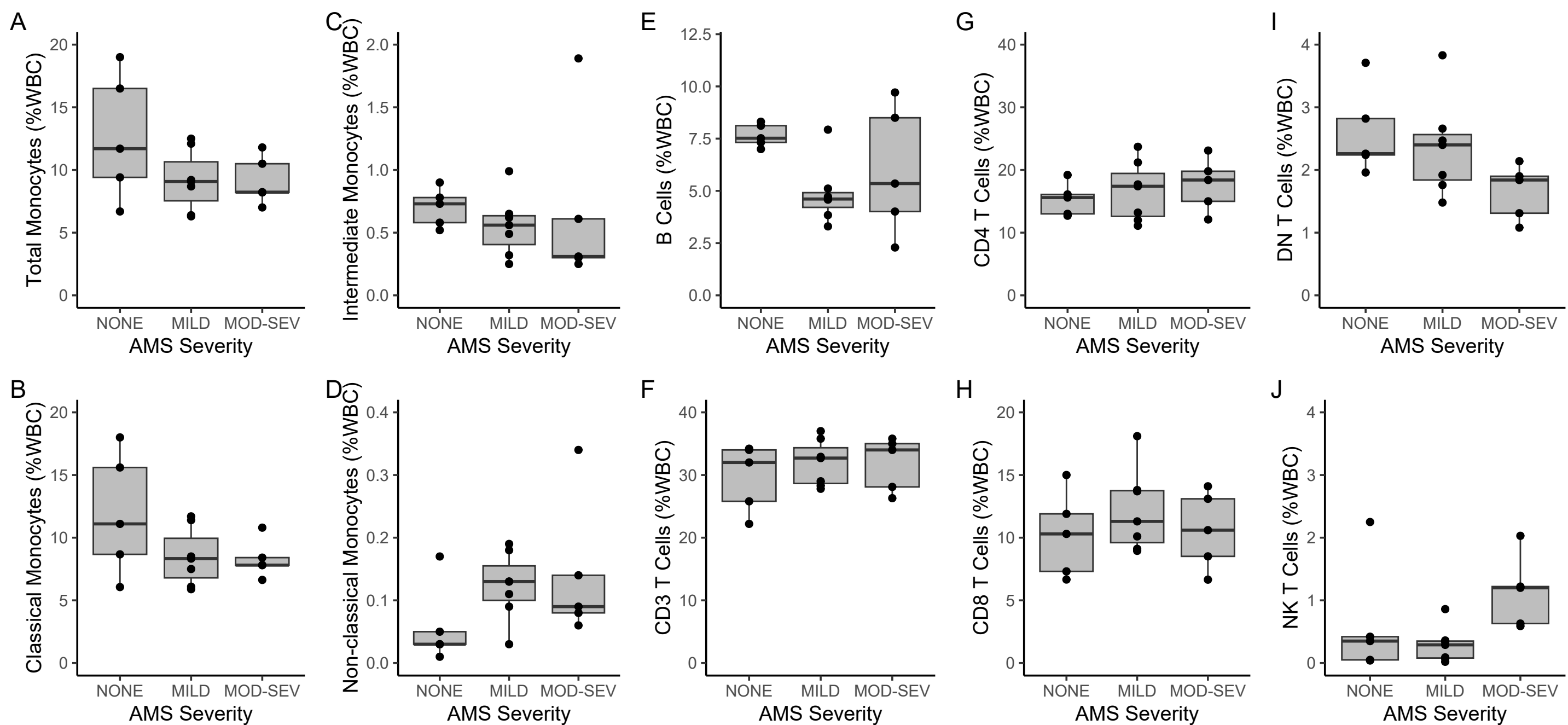

Supplement: Supplementary file 8 — Figure S8. Relationships between sea level baseline immune cell populations and AMS severity on the first day at high altitude. Upper and lower box limits correspond to the first and third quartiles, thick center lines represent medians, and outliers outside 1.5 * IQR are represented as unconnected points. Post‐hoc pairwise t‐test p values are provided for groups showing significant main effects of location via one‐way ANOVA. AMS severity groups: None (0–2), Mild (3–5). Moderate–Severe (6+). [file PHY2-12-e70024-s008.pdf]
